# Biopolymeric Films and Coatings Based on Purple Corn Flour and Propolis: Physicochemical Properties and Application in the Preservation of Fuerte Avocado

**DOI:** 10.3390/polym18030417

**Published:** 2026-02-05

**Authors:** Ronald Díaz-Saenz, Dagnith L. Bejarano-Luján, Franklin Lozano, Luis R. Paredes-Quiroz

**Affiliations:** 1 Facultad de Ingeniería, Universidad Nacional Micaela Bastidas de Apurímac, Abancay 3001, Peru; 232434@unsaac.edu.pe (R.D.-S.); flozano@unamba.edu.pe (F.L.); 2 Facultad de Ingeniería, Universidad Nacional Autónoma de Tayacaja Daniel Hernández Morillo, Pampas 09156, Peru; lizbejarano@unat.edu.pe; 3 Grupo de Investigación, Ciencia e Innovación para el Desarrollo Integral de la Agroindustria, Universidad Nacional Micaela Bastidas de Apurímac, Abancay 3001, Peru

**Keywords:** purple corn flour, ethanolic propolis extract, edible films, biodegradable coatings, shelf life, physicochemical properties

## Abstract

Natural preservation technologies have emerged as sustainable alternatives for maintaining the postharvest quality of fresh products. This study developed and characterized edible films and coatings produced from purple corn flour (MMH) and ethanolic propolis extract (EEP), and evaluated their effectiveness in extending the shelf life of Fuerte avocado. Film-forming solutions were prepared using three MMH/EEP formulations (100/0, 90/10, and 80/20), and their apparent viscosity was determined. Films obtained by drying at 45 °C for 12 h were analyzed for pH, thickness, tensile strength, solubility, water vapor permeability, and microstructure by SEM. The MMH 80/20 EEP formulation showed the best overall performance and was selected as a coating for avocados stored under ambient and refrigerated conditions. Shelf life was defined based on quantitative criteria, including acceptable limits of weight loss and sensory acceptability. Under these criteria, coated avocados reached a shelf life of 30 days at ambient temperature, compared to 15 days for uncoated fruit, and 72 days under refrigerated storage, compared to 50 days for the control. Additionally, the coating reduced weight loss, preserved moisture, and improved sensory acceptance. Overall, MMH/EEP systems represent a promising natural alternative for the postharvest preservation of avocado.

## 1. Introduction

The avocado (Persea americana), a highly valued tropical–subtropical fruit worldwide, has shown a remarkable increase in production in response to the growing demand of the global market [1,2]. Countries such as Mexico, Chile, the United States, and Peru lead global production. However, challenges in management practices, as well as in production and commercialization processes, lead to significant losses during harvest and post-harvest stages, negatively affecting the efficiency and profitability of the crop [3,4,5].

Postharvest waste represents the largest component of food loss, driving research and development of edible coatings and films as an innovative alternative for food packaging. These technologies aim to improve the preservation of perishable products by extending their shelf life, thus addressing a critical challenge within the food supply chain [6,7]. However, these technologies still face significant barriers in the optimization of preservation properties, which are essential for maintaining the nutritional and organoleptic quality of foods [7,8].

The current focus of research on edible coatings has extended beyond the simple use of these materials, exploring innovative packaging systems that not only prolong the shelf life of food but also reduce susceptibility to microbiological spoilage and oxidation processes, key elements for maintaining freshness and food safety [9,10]. Edible films and coatings contribute to product protection against factors such as weight loss, firmness, transpiration, and dehydration, and improve sensory characteristics such as color, shine, and peel flavor [11,12].

For the formulation of edible films and coatings, it is essential to evaluate the physicochemical properties of the base ingredients, such as polymers, lipids, and plasticizers—which determine the functionality and performance of the film [13]. The mechanical and physical characterization of these formulations is crucial for selecting the most appropriate application method, such as the dipping process [4,14].

Purple corn starch has emerged as a promising biopolymer in various industries, including food and pharmaceuticals, thanks to its abundance, affordability, biodegradability, and excellent hydrophilicity. However, the direct application of starch in films is limited due to its insufficient mechanical and barrier properties, requiring modifications to improve its functionality [15,16].

Propolis, a product derived from the beehive, is recognized for its complex chemical composition and broad spectrum of biological properties, including antibacterial, antifungal, antiviral, and antioxidant effects, among others. Research focused on developing new propolis-based materials from diverse geographical origins is essential for controlling pathogenic diseases and expanding its application in active packaging [17,18].

The literature reports that edible films and coatings formulated from polysaccharides, proteins, lipids, and composite biopolymers can effectively protect foods against oxidation, aroma loss, and lipid absorption. However, their practical application is often limited by high water vapor permeability and, in many cases, insufficient mechanical strength. To overcome these limitations, the incorporation of antimicrobial and antioxidant agents has been widely explored; nevertheless, several studies have demonstrated that although these additives enhance bioactive functionality, they may compromise the mechanical integrity of the material, leading to a reduction in tensile strength [19,20]. Only in specific systems, depending on the polymer matrix and additive concentration, have significant mechanical improvements been reported [21]. Consequently, recent research has emphasized reinforcement strategies, such as polymer blending or the incorporation of nanomaterials (e.g., cellulose nanocrystals), to improve mechanical and barrier properties. However, these approaches involve challenges related to process complexity, cost, and scalability. In this context, exploring synergistic interactions between biopolymeric matrices and natural bioactive compounds emerges as a promising yet still underexplored strategy.

The present study focused on the development of edible coatings and films based on purple corn flour, propolis, and the combination of both biopolymers. A detailed characterization was carried out to identify the most suitable formulation for application by dipping on Fuerte avocado, with the aim of extending its shelf life under ambient and refrigerated storage conditions.

To contextualize the study, a comparative table is included summarizing the typical performance, advantages, and limitations of biopolymers used in edible films and coatings (Table 1), highlighting the main technological constraints addressed in the present research.

## 2. Materials and Methods

### 2.1. Materials

Fuerte avocados, propolis, and purple corn were obtained from the rural community of Supalla, Aymaraes, Apurímac (2260 m a.s.l.), Peru. For the study, avocados of the same cultivar, at an appropriate maturity stage and with a 0.5 cm long peduncle, were selected and divided into two storage groups: ambient temperature (90 units) and refrigeration (117 units). The ethanolic extract of propolis (EEP) was obtained following the method described by Bankova et al. [30], with modifications. The propolis collected by scraping was subjected to successive extraction with 96% ethanol (*v*/*v*) for 7 days at room temperature and in the absence of light; during this period, manual agitation was performed daily for 30 min to promote homogenization. The mixture was then centrifuged at 3000 rpm for 10 min and filtered by gravity. The filtrate was concentrated in an oven (MEMMERT UN30) at 45 °C for 7 days until constant weight was achieved. The resulting EEP resin was stored in amber containers to prevent oxidation and kept refrigerated until use. Purple corn kernels, previously separated from the pericarp, were ground and sieved using a RETSCH AS200 system equipped with a No. 170 mesh (90 μm) to obtain purple corn flour (MMH). The research was conducted in the Chemistry and Sensory Analysis laboratories of the Universidad Nacional Micaela Bastidas de Apurímac (UNAMBA).

### 2.2. Physicochemical Analysis of EEP and MMH

The ash and moisture percentages of both products were determined gravimetrically, following the official AOAC methods: Method 942.05 for ash and Method 925.10 for moisture [31]. The insoluble material content was evaluated according to the methodology described by Bankova et al. [30], with modifications. For this purpose, 2.25 g of EEP were weighed into a beaker, and 100 mL of 96% ethyl alcohol were added, stirring the mixture continuously for 24 h until complete dissolution was achieved. Subsequently, the solution was then filtered using Whatman No. 1 filter paper, and the retained residue was dried in a MEMMERT UN30 oven at 110 ± 2 °C for 2 h. The percentage of insoluble material was calculated using the following equation:

(1) $$Insoluble\;material(\%)=\frac{P_{3}-P_{1}}{P_{2}}\;\times \;100$$

where: *P*_1_ is the weight of the empty filter paper (g); *P*_2_ is the initial dry weight of the EEP sample (g); *P*_3_ is the weight of the filter paper plus the insoluble residue after drying (g).

### 2.3. Extraction and Determination of Total Anthocyanins in MMH

The extraction of anthocyanins from purple corn flour (MMH) was carried out following the procedures described by Gorriti et al. [32] and Barragan et al. [33], with modifications. A total of 2.5 g of purple corn cob, previously ground and sieved to a particle size of Ø 1 mm, were extracted with 200 mL of a 20% (*v*/*v*) hydroalcoholic ethanol solution. The extraction solvent was acidified and adjusted to pH 2.0 by the addition of concentrated HCl, and the extraction was carried out at 90 °C for 120 min. The resulting extracts were filtered using Whatman No. 1 filter paper under vacuum with a COPELAMETIC filtration system. An aliquot of the extract was diluted to 25 mL in a volumetric flask using potassium chloride buffer (pH 1.0) and sodium acetate buffer (pH 4.5). Total anthocyanin content was determined by the differential pH method described by Giusti et al. [34], using cyanidin-3-glucoside as the reference pigment. Absorbance measurements were recorded at 510 and 700 nm on a GENESYS 10S UV-VIS spectrophotometer (Espectronic, USA), using ultrapure water as the blank.

Differential absorbance (A) was calculated as follows:

(2) $$A=\left(A_{510}-A_{700}\right){pH}_{1.0}-\left(A_{510}-\;A_{700\;}\right){pH}_{4.5}$$

The concentration of monomeric anthocyanins was calculated using the equation:

(3) $$CAT\left(\frac{mg}{L}\right)=\;\frac{A\;.\;\;PM\;.\;\;FD.\;\;1000}{\varepsilon .l}$$

where CAT (mg/L) is the total anthocyanin content; A is the differential absorbance; **PM** is the molecular weight of cyanidin-3-glucoside (449.2 g·mol^−1^); FD is the dilution factor; ε is the molar absorptivity coefficient (26,900 L·mol^−1^·cm^−1^); and l is the path length of the cuvette (1 cm).

Finally, the pH was measured using an S1 ANALYTICS LAB 850 pH meter, calibrated according to the manufacturer’s specifications.

### 2.4. Preparation of Edible Films from Purple Corn Flour and Purple Corn Flour with Ethanolic Propolis Extract (MMH and MMH/EEP)

MMH and MMH/EEP films were prepared using the casting method. The composition of the different formulations is presented in Table 2. Aqueous MMH solutions at 3% (*w*/*w*) were prepared using glycerol as a plasticizer at 66.67% (*w*/*w*) relative to the MMH mass, employing a magnetic stirrer (SPINBAR 1” × 5/16”). The film-forming solutions were then heated to 80 °C for 10 min under continuous stirring, and subsequently cooled to 45 °C for 12 h at a relative humidity of 60%.

The EEP was incorporated at two concentrations (10% and 20% *w*/*w*, based on the MMH mass), selected according to preliminary formulations previously evaluated (MMH/EEP 50/50, 60/40, 70/30, 80/20, and 90/10). To ensure homogeneous dispersion of the EEP, both solutions were further homogenized using magnetic agitation. Then, 20 mL of each formulation were poured into 10 cm glass Petri dishes previously lined with Teflon tape, and dried in an oven (MEMMERT UN30) at 45 °C for 12 h under controlled relative humidity (60%) to allow solvent evaporation. Once dried, the films were placed in a desiccator to prevent moisture absorption. The resulting samples were labeled as MMH 100% (control), MMH 90%/EEP 10%, and MMH 80%/EEP 20%.

### 2.5. Viscosity Measurement of Film-Forming Solutions

The viscosity of the MMH and MMH/EEP film-forming solutions was determined at 45 °C using a Brookfield viscometer (BROOKFIELD CERTIFIED 8664565, USA), equipped with spindle No. 4 and operated at speeds ranging from 10 to 50 rpm.

### 2.6. Physical and Mechanical Properties of the Edible Films

The water vapor permeability of the edible films was evaluated according to ASTM standard E96/E96M-05 [35], which establishes a maximum mass variation limit of 10% relative to the initial weight. Film thickness and tensile strength were determined following ASTM D882-12 [36]. Film solubility was measured according to the method described by Wang et al. [37], with modifications. For this analysis, the films were cut into 2.5 cm diameter discs and dried in a MEMMERT UN30 oven at 100 °C for 6 h until a constant weight was reached. The samples were then immersed in 100 mL of distilled water for 24 h. At the end of this period, the film residues were recovered by decantation and dried again at 90 °C until a constant weight was reached. The soluble fraction was expressed as a percentage of mass loss, calculated using the equation:

(4) $$Weight\;Loss\;(\%)=\frac{P_{i}-P_{f}}{P_{i}}\;\times \;\;100$$

where P_i_ is the initial weight of the film sample (g), P_f_ is the final weight of the film sample after drying (g).

### 2.7. Scanning Electron Microscopy

The films were coated with a thin layer of gold by sputtering and subsequently analyzed using a scanning electron microscope (FEI QUANTA 650 SEM) at the Materials Characterization Center of the Pontifical Catholic University of Peru (PUCP).

### 2.8. Preparation of Avocados and Application of Film-Forming Solutions

The avocados were first disinfected by immersion in a 1% sodium hypochlorite solution for 5 min. After disinfection, the fruits were allowed to dry at ambient temperature and, once dry, were fully immersed in the film-forming solutions for 1 min at room temperature. In parallel, an uncoated control group was prepared. All samples were stored under two conditions: refrigeration at 6 °C for 72 days and ambient temperature (20 ± 2 °C) for 35 days.

### 2.9. Physicochemical Characteristics of Coated Fuerte Avocado

Fuerte variety avocados, coated with the MMH 80%/EEP 20% formulation, along with the uncoated control group, were evaluated at intervals of 0, 5, 10, 15, 20, 25, 30, and 35 days under ambient storage conditions (20 ± 2 °C), and from day 0 up to 72 days under refrigeration (6 °C). All physicochemical determinations were performed in triplicate. The pH was measured according to the methodology established by AOAC [38]. Texture was assessed following ASTM Specification D5 using a penetrometer (KOEHLER Penetrometer, K95590) from the Faculty of Chemistry and Metallurgy at the National University of San Antonio Abad del Cusco. Titratable acidity was determined by neutralization with 0.1 N NaOH in accordance with the Colombian Technical Standard NTC 4103. The oil content was quantified using a lipid extraction procedure following the method described by Tan et al. [39].

### 2.10. Sensory Evaluation

**Figure 5:**
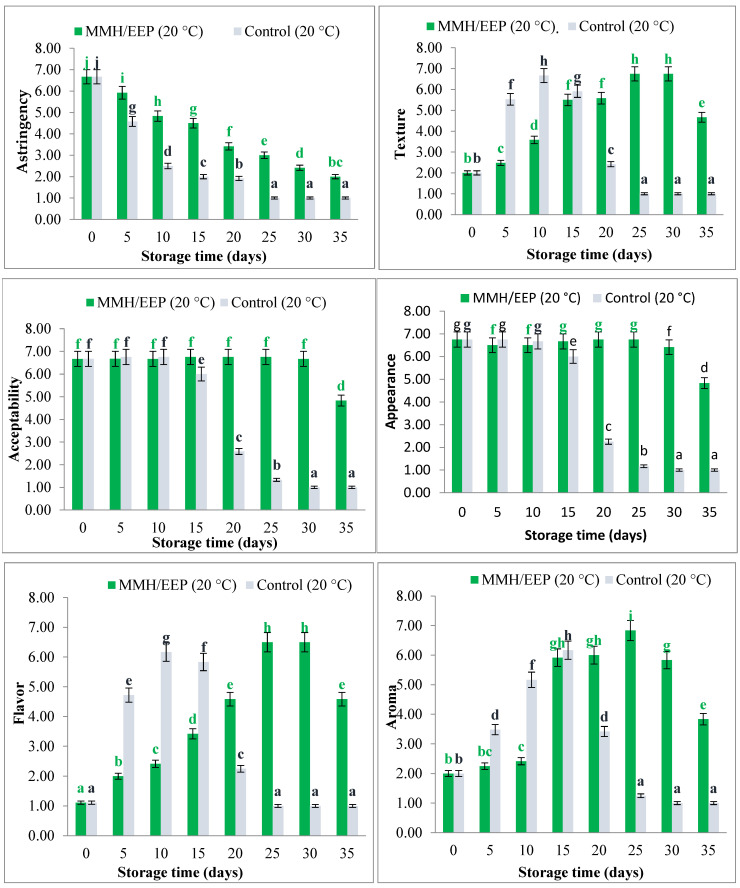
Sensory evaluation of astringency, texture, appearance, flavor, aroma, and overall acceptability of avocados treated with MMH 80%/EEP 20% and the control at 20 °C.

Fuerte avocados coated with the MMH 80%/EEP 20% formulation, along with the uncoated control group, stored at ambient temperature (20 ± 2 °C) and under refrigeration (6 °C), were subjected to sensory evaluation after 30 and 72 days of storage. Although sensory evaluations were conducted at both storage temperatures and time points, only the results obtained at ambient temperature up to 35 days are presented in Figure 5. This decision was made because avocados stored under refrigeration did not reach an adequate ripening stage for reliable sensory assessment within the evaluated period, whereas samples stored at ambient temperature exhibited clearer sensory changes relevant to consumer acceptance. Therefore, sensory observations from refrigerated storage and extended storage times are discussed qualitatively but were not included in the graphical analysis.

The analysis was performed in triplicate using a linear hedonic scale, with the participation of 36 untrained panelists between 20 and 30 years of age. Each avocado was cut into two halves and then divided into six longitudinal slices for individual assessment. To determine consumer acceptance, the mean values and standard deviations of the sensory scores were calculated. The hedonic scale enabled classification of product acceptability by assigning numerical values to each evaluated attribute, such as astringency, texture, appearance, flavor, aroma, and overall acceptability, following the methodology described by Watts et al. [40].

### 2.11. Statistical Analysis

Statistical analysis was performed using one-way analysis of variance (ANOVA) under a completely randomized design, employing R software (version 4.1.3). Experimental results were reported as the mean ± standard deviation (SD) of three replicates. When significant differences were detected (*p* < 0.05), Tukey’s multiple comparison test was applied to identify differences among treatments.

## 3. Results and Discussion

### 3.1. Physicochemical Characterization of EEP and MMH

It was observed that the ash content was slightly lower in MMH (0.16%) compared with EEP (0.20%), while the moisture content was higher in MMH (12.86%) regarding the EEP (7.28%). The percentage of insoluble material in EEP was significant, reaching 21.19% (Table 3). These values coincide with trends reported in the literature. For example [41], analyzed purple corn flour and found moisture values between 9.88% and 11.40%, with an ash content of approximately 1.45%. Similarly [42], reported moisture values of 11.70% and ash content of 0.02% for purple corn flour. Both studies attributed compositional variations to factors such as environmental growing conditions, temperature, cultivar, and seed type (yellow, white, black, blue, or purple).

Regarding propolis [43], reported moisture contents of 3.5% and ash contents of 1.2% in Moroccan propolis, associating these variations with geographical origin and harvesting conditions. The results obtained in the present study fall within the expected range of variability for natural matrices influenced by environmental and botanical differences.

Regarding the purple corn flour, the results obtained in this study are consistent with those reported by Vilcacundo et al. [42], who documented a moisture content of 11.70%, and are slightly below the values described by Sukainah et al. [44], who reported 16.1% moisture and 0.25% ash in corn flours. These values fall within the parameters established by the Peruvian Technical Standard 209.027:2016 [45]., indicating that the raw material used in the present study exhibited appropriate storage and preservation conditions.

The total anthocyanin content determined in the purple corn flour was lower than that reported by Ccaccya et al. [46], who reported concentrations between 24.4 and 42.6 mg of cyanidin-3-glucoside/g in cobs from different purple corn varieties collected in Abancay, Cajamarca, and Lima. Similarly, [47] evaluated three purple corn varieties from the Cajamarca region and highlighted the INIA-601 variety, which exhibited a yield of 4.38 t/ha and anthocyanin contents of 7.9 mg/g dry matter in the cob and 4.53 mg/g dry matter in the bracts, values lower than those obtained in our study (9.17 mg/g dry matter). Likewise, [48] reported anthocyanin levels of 0.85 mg/g in the grain and 9.59 mg/g in the pericarp of purple corn, following the same analytical methodology used in the present study, complemented with ultrasound-assisted extraction.

The observed differences can be attributed to the part of the plant material used. In particular, the higher anthocyanin contents reported by Ccaccya et al. [46] would be justified because the cob is one of the structures where the greatest amount of anthocyanins is concentrated, compared to the grains or external fractions of the corn, as also highlighted by Tepixtle-Colohua et al. [48] and Bustamante-Bernedo et al. [49].

### 3.2. Viscosity of Film-Forming Solutions

**Figure 1:**
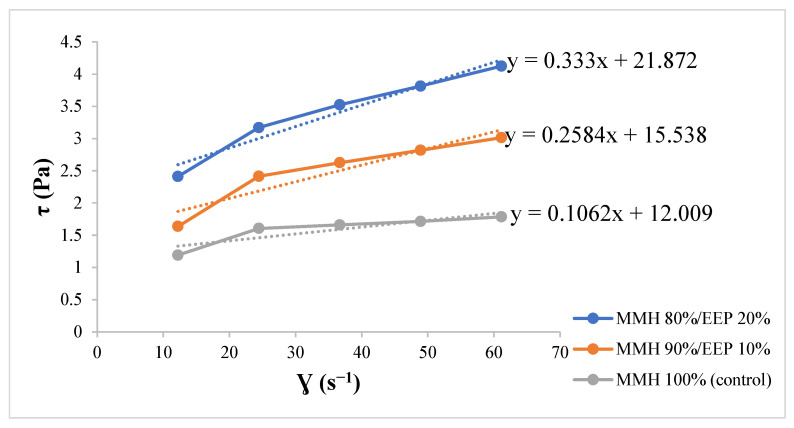
Flow curves of film-forming solutions: MMH 100% (control), MMH 90%/EEP 10%, and MMH 80%/EEP 20%. The curves illustrate the non-Newtonian pseudoplastic behavior of the systems. Model fitting was evaluated using the coefficient of determination (R^2^), without statistical comparison of model equations.

Figure 1 shows the flow curves of the film-forming solutions: MMH 100% (control), MMH 90%/EEP 10%, and MMH 80%/EEP 20%. All formulations exhibited nonlinear flow curves that did not pass through the origin, confirming a typical pseudoplastic behavior. Based on the coefficient of determination (R^2^) values presented in Table 4, the Ostwald–de Waele model was selected as an appropriate descriptive model to compare the rheological behavior of the MMH/EEP systems.

The incorporation of EEP increased the shear stress required to deform the system, with higher EEP concentrations leading to higher shear stress values. Accordingly, the apparent viscosity increased with EEP addition, ranging from 0.04544 Pa·s for MMH 90%/EEP 10% to 0.05709 Pa·s for MMH 80%/EEP 20%, measured at a shear rate of 61.15 s^−1^.

The Ostwald–de Waele model was applied as a comparative descriptive approach to evaluate flow behavior among formulations. Model adequacy was assessed using the coefficient of determination (R^2^). The consistency index (K) and flow behavior index (n) were not reported, as the objective of this study was not to establish a complete rheological model but rather to compare the pseudoplastic behavior of the film-forming solutions.

These findings are consistent with those reported by Vergel-Alfonso et al. [50], who developed and characterized coatings based on pectin and beeswax. The authors observed that beeswax concentration exerted a slight effect on shear stress and viscosity of the mixture: a higher beeswax content increased the shear stress required for the same strain rate and reduced the apparent viscosity, confirming pseudoplastic rheological behavior. Similarly, the MMH/EEP film-forming solutions evaluated in the present study exhibited pseudoplastic behavior, with comparable flow curves for the same strain rate profile. These curves allowed for the precise characterization of the rheological nature of the formulations, establishing the relationship between strain rate and the material’s response under a given shear rate. Likewise, [51] highlight that the viscosity of a film-forming system is influenced not only by solute concentration but also by properties such as plasticity, smoothness, stickiness, particle size, and temperature, features that were evident in the solutions analyzed in this study. Overall, these rheological attributes support the suitability of the MMH/EEP formulations for application using the dipping method.

The film-forming solution composed of both biopolymers (purple corn flour and ethanolic propolis extract in an 80:20 ratio) exhibited high structural cohesion and markedly improved the mechanical and barrier properties when applied to the surface of Fuerte avocado. This formulation also demonstrated strong adhesion capacity, contributing to greater stability and durability of the edible coating during storage. In contrast, films made with 90:10 and 100:0 ratios of MMH/EEP and pure MMH, respectively, showed higher permeability, possibly associated with their greater porosity, flexibility, and structural fragility.

The viscosity of the film-forming solutions plays a critical role in the coating process. Solutions with low viscosity tend to promote rapid phase separation, resulting in thin and non-uniform films; conversely, higher viscosities reduce this separation, although excessively high viscosity may lead to films that are too thick for proper application. During immersion application, two main forces develop: cohesion between the coating molecules and adhesion between the fruit surface and the resulting film [52]. The combination of these biopolymers therefore demonstrates high potential for applications in the food industry, where the balance between cohesion, adhesion, and shear strength is essential to ensure the protection and extended shelf life of fresh produce.

### 3.3. Physical Characteristics of the Films

The pH, solubility, thickness, and permeability values of the films decreased significantly (*p* < 0.05) when 10% and 20% EEP were incorporated into the MMH matrix (Table 5). Similar behavior was reported by Mendoza-Intriago et al. [53], who observed that treating avocado fruit with alcoholic extract of propolis (30%) produced slight variations in pH. However, the authors emphasized that, despite the statistical differences compared to the control, propolis did not substantially modify the normal pH of avocado, whose average value is 6.24. These results agree with those found in our study, where no relevant differences in pH were observed between the MMH and MMH/EEP films, suggesting that the incorporation of EEP does not significantly alter the acidity of the film-forming system or its potential interaction with the coated fruit.

De Carli et al. [54] observed that the incorporation of different proportions of EEP (5%, 10%, and 20%) progressively increased the thickness of chitosan films, yielding values of 66.7 ± 8.2 μm, 70.0 ± 8.9 μm, and 71.7 ± 7.5 μm, respectively, with no significant differences among them (*p* > 0.05). The authors attributed this increase to the various interactions established between chitosan and the components of EEP. This behavior contrasts with the results obtained in our study, where the addition of EEP led to a reduction in film thickness. This effect, however, coincides with that reported by El-Sakhawy et al. [55], who reported that the incorporation of ethanolic propolis extracts into polysaccharide matrices can alter the morphology during the drying process, reduce film thickness and enhance polyphenol–polysaccharide interactions, which is reflected in greater tensile strength. These authors also emphasize that the effects of EEP depend on the polymer matrix used, the concentration incorporated, and the processing conditions.

The MMH and MMH/EEP films showed significant differences (*p* < 0.05) in their water resistance. Films produced solely with MMH exhibited higher solubility, whereas the incorporation of EEP progressively reduced this parameter, indicating greater stability against water exposure. Reference [56] attributes these variations to the physicochemical properties derived from the chemical structure of the components used in the formulation, which directly influence the molecular organization of the polymeric matrix by promoting intermolecular interactions, particularly hydrogen bonding between the hydroxyl groups of the polysaccharide chains and the phenolic compounds present in the ethanolic propolis extract. These interactions reduce chain mobility and lead to a more compact and ordered structure, which explains the observed decrease in water vapor permeability of the MMH-based films after EEP addition. Recent studies have shown that ethanolic propolis extracts can slightly reduce water vapor permeability in polysaccharide films, such as pectin films or those containing added hydrophobic components, an effect associated with both the hydrophobic resinous constituents of propolis and the microstructural modifications induced in the polymer matrix [57].

### 3.4. Mechanical Properties of the Films

**Figure 2:**
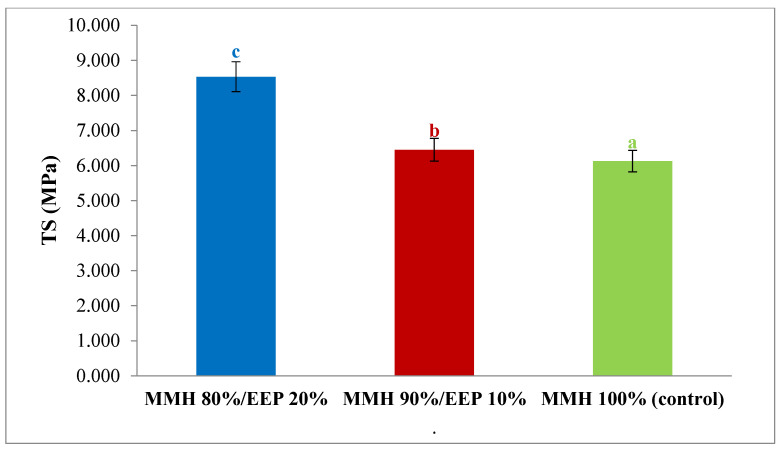
Tensile strength of films prepared with MMH 100% (control), MMH 90%/EEP 10%, and MMH 80%/EEP 20%. Values are expressed as mean ± standard deviation. Different lowercase letters indicate significant differences among formulations (*p* < 0.05), according to Tukey’s post hoc test. Elongation at break was not evaluated; tensile strength was used as an indicator of mechanical resistance related to handling and coating application.

The mechanical resistance results of the films prepared with MMH and EEP showed significant differences (*p* < 0.05) (Figure 2). The incorporation of 20% EEP increased tensile strength by 39.36% compared with the MMH film, while the addition of 10% EEP produced a slight but consistent improvement relative to the control. Elongation at break was not evaluated in this study; therefore, the mechanical characterization focused on tensile strength as an indicator of film resistance, which is a key parameter related to handling and structural integrity during coating application.

Although elongation at break is an important parameter associated with film flexibility, previous studies have reported that polysaccharide-based films incorporating phenolic compounds often exhibit increased rigidity accompanied by reduced extensibility. Consequently, the present results provide a comparative assessment of mechanical reinforcement rather than a complete flexibility characterization. These findings are consistent with those reported by Junior et al. [58], who observed that the incorporation of 3% EEP into sodium alginate films increased tensile strength from 12.9 to 16.5 MPa. In contrast, study [59] reported that films based on apple pulp pectin and pea protein supplemented with 3% propolis extract did not show significant differences in tensile strength compared to the control, whereas the incorporation of 12% EEP resulted in a significant decrease. This behavior was attributed to the hydroxyl groups of phenolic compounds acting as plasticizing agents, weakening primary polymer–polymer interactions and promoting phenol–protein or phenol–pectin interactions, leading to more flexible but less resistant films.

In contrast, [55] reported that incorporating ethanolic propolis extracts into polysaccharide matrices can alter film morphology during drying, reducing film thickness and strengthen polyphenol–polysaccharide interactions, thereby increasing tensile strength. These effects depend on the matrix used, the concentration of the extract, and the processing conditions. Regarding the role of concentration, [60] indicate that at high levels, propolis can form lipophilic domains or act as a local plasticizer, weakening the polymer matrix. While at low or moderate concentrations the interaction effect predominates, in which the phenolic compounds of propolis (flavonoids, phenolic acids, among others) can establish hydrogen bonds or specific interactions with polysaccharides and proteins of purple corn, increasing rigidity, cohesion, and consequently, tensile strength.

This dual behavior of propolis may explain why some studies report increases and others decreases in mechanical resistance, depending on the polymeric matrix and concentration range evaluated. The improved mechanical performance observed in MMH/EEP composite films may be associated with favorable interactions between the polysaccharide matrix and phenolic compounds present in the ethanolic propolis extract. Although no direct physicochemical characterization such as FTIR or X-ray diffraction was conducted in this study, the combined enhancement of tensile strength and barrier properties suggests a modification in the polymer network organization. Similar behavior has been reported in polysaccharide-based films containing polyphenolic compounds, where hydrogen bonding and structural rearrangements are proposed as contributing factors. However, further spectroscopic and structural analyses would be required to elucidate the exact nature of these interactions.

Elongation at break (EAB) was not evaluated in this study; however, polysaccharide-based edible films typically exhibit EAB values ranging from 10% to 60%, depending on the polymer matrix and plasticizer content. Numerous studies indicate that an increase in tensile strength is often accompanied by a reduction in EAB, reflecting the classical strength–flexibility trade-off. Therefore, the determination of EAB is proposed as a key parameter in future studies to complete the mechanical characterization of MMH/EEP films.

Although the increase in tensile strength suggests enhanced interactions between purple corn flour polysaccharides and propolis polyphenols, the absence of spectroscopic or diffraction analyses, such as FTIR or XRD, represents a limitation of the present study. These techniques would allow direct confirmation of molecular interactions and structural organization within the polymeric system and are therefore proposed as complementary tools for future studies aimed at further elucidating the structure–property relationships of these edible coatings.

Although elongation at break (EAB) is a relevant mechanical parameter for polymeric films, it was not evaluated in the present study. The mechanical characterization focused primarily on tensile strength, which is more directly related to the structural integrity and handling resistance required for edible coatings intended for postharvest fruit preservation. In this context, flexibility was considered a secondary parameter compared to barrier performance and mechanical stability. The evaluation of EAB will be addressed in future studies to provide a more comprehensive mechanical characterization of the films.

### 3.5. Film Morphology

**Figure 3:**
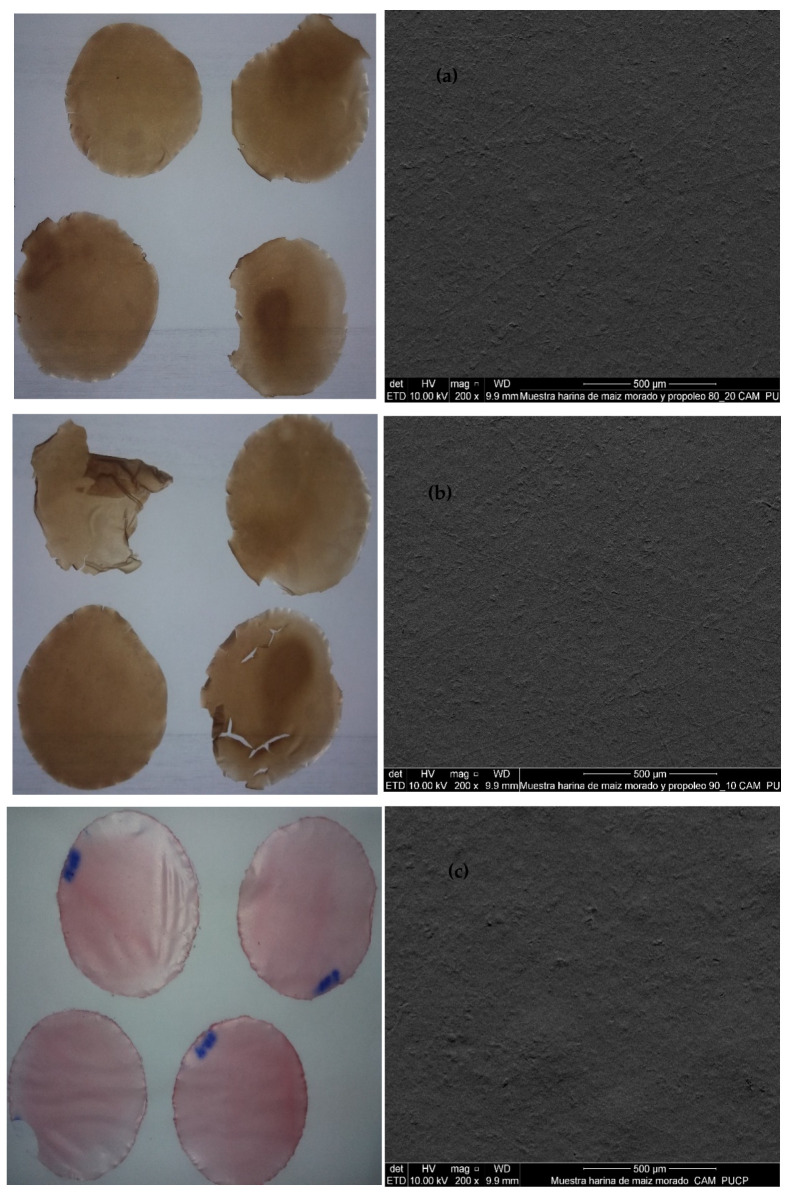
Surface SEM micrographs of edible films prepared from purple corn flour (MMH) and ethanolic propolis extract (EEP): (**a**) MMH 80%/EEP 20%, (**b**) MMH 90%/EEP 10%, and (**c**) MMH 100% (control). Films containing EEP show a more homogeneous polymeric matrix with reduced aggregation compared to the control. The SEM analysis was qualitative, and no particle size distribution was determined.

From a qualitative perspective, Figure 3 shows surface micrographs of films made from purple corn flour and ethanolic propolis extract (MMH/EEP). SEM micrographs suggest a more homogeneous distribution of the polymeric matrix in films containing EEP. Although a clear quantitative reduction in insoluble particles cannot be conclusively established from the micrographs, the presence of EEP appears to promote improved matrix continuity and reduced aggregation tendencies compared to MMH films without EEP. This reduction suggests improved integration and compatibility between the film components at higher EEP concentrations.

Similar behavior was reported by Cunha et al., 2021 [61] in cassava starch films supplemented with propolis extract, where residual starch particles dispersed within the matrix were observed. According to the authors, these particles may act as discontinuities in the film structure, affecting its uniformity and certain physical properties. The results obtained in the present study are consistent with this trend, indicating that EEP may contribute to a more homogeneous matrix and a reduction in surface defects.

The incorporation of EEP into the MMH matrix resulted in more compact and less fragile films. The MMH 80%/EEP 20% formulation exhibited easier detachment from the Petri dish and showed lower fragility than the MMH 90%/EEP 10% and control films, exhibiting a uniform matrix, smooth surface, good flexibility, and homogeneously distributed particles, which reflects a more continuous and cohesive material. The SEM images of MMH-based films without EEP exhibit a less uniform surface morphology, characterized by localized irregularities and heterogeneities. While features such as pores or discontinuities are observed, these characteristics are discussed qualitatively, as SEM provides descriptive rather than quantitative information regarding film fragility or porosity. In contrast, films produced solely with MMH showed greater fragility, the presence of air bubbles, small lumps, a rough texture, and noticeable porosity, possibly associated with their high water vapor permeability, as suggested by Wang et al. [62]. The addition of ethanolic propolis extract markedly improved the structural cohesion of the film, presumably due to interactions between the polysaccharides in purple corn flour and the phenolic compounds present in propolis. Similar behavior has been reported in pectin-based films supplemented with green propolis extract [60]. These findings also coincide with those reported by Akkuzu et al. [63], who observed that interactions between chitosan and propolis in edible films can strengthen interfacial adhesion, tighten chain-to-chain interactions of the polymer, and consequently improve its mechanical strength.

Several studies have demonstrated that the addition of propolis extract to polymeric matrices enhances film homogeneity, due to the presence of hydroxyl groups in the phenolic compounds of propolis [61]. These authors indicate that such hydroxyl groups can form hydrogen bonds with the hydroxyl groups of starch, thereby reducing intermolecular interactions between polymer chains and improving the structural uniformity of the film. Furthermore, increasing the concentration of propolis extract in cassava starch films was shown to decrease surface roughness, reflecting improved compatibility between the propolis constituents and the cassava starch matrix.

In the present study, the greater homogeneity observed in the MMH/EEP film matrix resulted in an increase in mechanical strength, an effect also reported by other authors [63]. Similarly, tensile strength increased with the incorporation of EEP, showing significant differences compared with the control (Figure 2). This behavior suggests that the integration of propolis in the formulation plays a decisive role in enhancing the physical and mechanical properties of the edible films. The MMH 80%/EEP 20% ratio not only promoted the formation of a more uniform and compact structure but also reduced surface and structural defects that could compromise the integrity of the coating during handling and use.

### 3.6. Physicochemical Characterization of Coated Avocados

Table 6 presents the physicochemical characterization results of coated and uncoated avocados. Statistically significant differences (*p* < 0.05) were observed in titratable acidity, moisture content, oil content, texture, pH, and weight loss among treatments, as well as across different storage times and temperatures.

On day 0, the control samples exhibited a pH of 6.13 and a titratable acidity of 0.04% citric acid. After 35 days of storage, uncoated avocados showed an increase in pH to 6.74 at 20 °C and 6.52 at 6 °C, while titratable acidity decreased to 0.01% and 0.03% citric acid, respectively. In the samples coated with MMH/EEP, the pH values on day 35 reached 6.65 at 20 °C and 6.30 at 6 °C. Titratable acidity remained at 0.01% at 20 °C and 0.03% citric acid at 6 °C. These findings are consistent with those reported by Mendoza-Intriago et al. [53], who observed pH values ranging from 6.19 to 6.23 in avocados treated with alcoholic propolis extract (30%) during 12 days of storage, values that closely resemble those obtained in the present study.

The coating, together with storage under both ambient and refrigerated conditions, helped maintain lower pH values compared with uncoated avocados. Regarding titratable acidity, a gradual decrease was observed in the coated avocados by the end of the experiment compared with day 0. These results indicate that the coating formulated with purple corn flour and ethanolic propolis extract was effective in delaying the ripening process.

Filgueiras et al. [25] note that the application of edible coatings and the use of low temperatures reduce the fruit’s respiration rate, decreasing the anabolic and catabolic activities characteristic of ripening. During this process, starch is hydrolyzed to glucose through glycolysis; subsequently, the Krebs cycle transforms pyruvic acid and other organic acids, producing CO_2_, acids, heat, and water. These metabolic transformations directly affect the values of pH, acidity, soluble solids and moisture content, which explains the variations observed in the analyzed physicochemical parameters.

**Figure 4:**
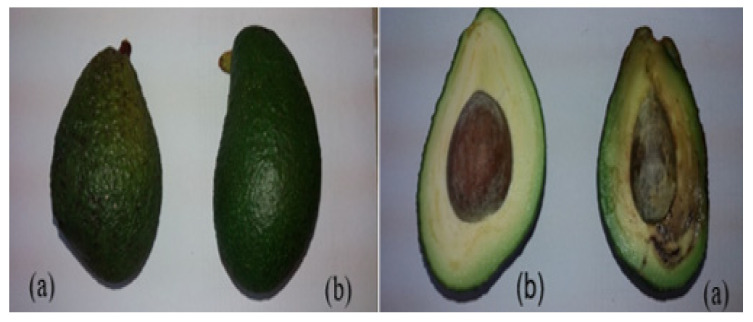

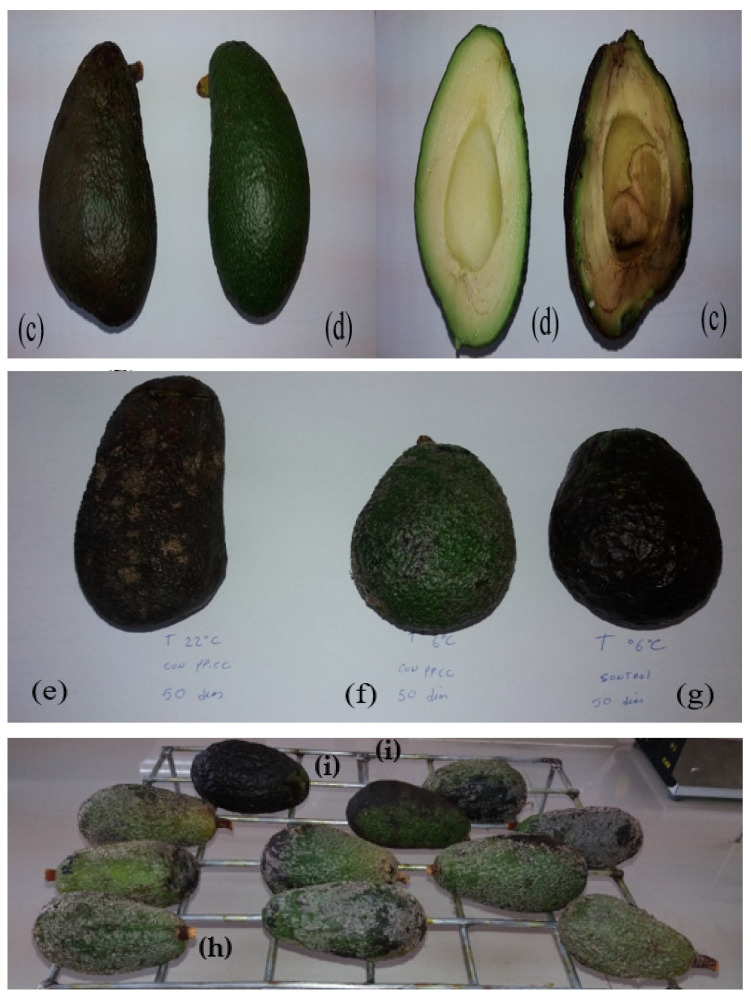
Visual appearance of Fuerte avocado samples without coating (control) and coated with a purple corn flour solution (MMH 80%) containing ethanolic propolis extract (EEP 20%) during storage. Avocados after 15 days of storage at ambient temperature (20 ± 2 °C): control (**a**) and coated (**b**); Avocados after 35 days of storage at ambient temperature: control (**c**) and coated (**d**); Avocados after 50 days: coated at ambient temperature (**e**); coated at 6 °C (**f**); control at 6 °C (**g**); Avocados after 72 days of storage at 6 °C: coated (**h**) and control (**i**).

Fuerte avocados stored at 6 °C did not reach an adequate stage of ripening, likely because this temperature is below the optimal range recommended for agro-industrial operations, which lies between 8 and 12 °C [64]. Coated avocados kept at 6 °C showed signs of dehydration, cracking, and partial detachment of the edible coating. In contrast, coated avocados stored at ambient temperature remained suitable for consumption for 25 to 30 days, whereas uncoated samples maintained acceptable quality for only 10 to 15 days. Under refrigerated conditions, coated avocados preserved their integrity for up to 72 days, compared with uncoated fruits, which lasted only up to 56 days and did not reach full ripening (Figure 4).

Although ‘Fuerte’ avocado is known to be susceptible to chilling injury at temperatures below 7 °C, the present study did not include specific physiological indicators of chilling injury such as electrolyte leakage or external skin browning. Therefore, shelf life under refrigerated conditions was defined based on visual integrity, weight loss, firmness evolution, and sensory acceptability rather than direct chilling injury markers, and the occurrence of subclinical chilling effects cannot be ruled out.

Texture analysis revealed significant changes in the recorded parameters, indicating the progressive softening of avocados during storage. This behavior is consistent with previous reports showing that edible coatings applied to horticultural products can modulate enzymatic activity associated with fruit softening by partially limiting gas exchange, thereby contributing to texture preservation [65]. The variations observed in texture values reflect a differentiated response of coated fruits to metabolic activity and moisture loss throughout the ripening process. This trend was evidenced by an increase in needle penetration depth measured by the texture analyzer, which is indicative of tissue softening. In contrast, avocados stored under refrigerated conditions did not reach an adequate ripening stage due to prolonged exposure to low temperatures, a behavior that has also been reported by Olivares et al. [66].

The percentage of titratable acidity decreased slightly during storage, in agreement with the findings of [67], who reported that this reduction is associated with the activity of dehydrogenase enzymes and the utilization of organic acids as respiratory substrates for the synthesis of new compounds during ripening. This decrease in acidity coincides with the onset of the ripening process and the rapid accumulation of sugars in the fruit. According to these authors, ripeness is characterized by an increase in total soluble solids and a progressive decrease in titratable acidity, particularly in climacteric fruits. This behavior occurs when the fruit reaches its maximum respiratory rate and rapidly consumes its reserves of organic acids as a consequence of increased metabolic activity. Complementarily, [68] suggest that the reduction in acidity may be related to the consumption of organic molecules in metabolic cycles for energy production, as well as to the transformation of various organic acids into volatile compounds, whose presence intensifies during the ripening process.

At the same storage temperature, no significant differences in titratable acidity were observed between coated and uncoated avocados after 35 days of storage. This behavior can be attributed to the fact that titratable acidity is not the most sensitive parameter to coating application, particularly at advanced storage times. During prolonged storage, organic acid metabolism may reach a relative equilibrium, and temperature becomes the dominant factor governing acidity changes. In this context, edible coatings mainly influence water loss and respiration rate, while their effect on titratable acidity may be limited, which is consistent with previous reports for coated climacteric fruits.

The moisture content of the avocados showed a slight decrease during storage; however, this reduction remained within ranges that did not compromise the appearance or commercial value of the fruit. Recent studies confirm that the application of edible coatings to avocados is an effective strategy to reduce moisture loss and extend shelf life. Reference [69] reported that fruits treated with composite coatings retained greater firmness after 28 days of cold storage (5 °C) followed by an additional 7 days at ambient temperature (23 °C) compared with untreated fruits; moreover, the authors associated firmness loss primarily with water loss, which is regulated by storage temperature. Complementarily, [70] indicated that even moderate water losses (on the order of 3–10%) can affect fruit texture and commercial value; therefore, reducing moisture exchange through coatings, together with controlling temperature and relative humidity, is a recommended postharvest practice. In the present study, uncoated avocados stored at ambient temperature experienced a moisture loss of 47.5% over a 30-day period, whereas avocados coated with MMH 80%/EEP 20% and stored at 6 °C exhibited a substantially lower loss of 16% over the same period, demonstrating the effectiveness of the coating. These results are comparable to those reported by Garcia et al. [5], who highlighted that methylcellulose coatings significantly reduced moisture loss in avocados; after 6 days of storage, coated fruits lost only 4 g/100 g of moisture, compared with 8 g/100 g in control fruits. In addition, coated fruits reached their maximum CO_2_ levels after 8 days, compared with 6 days for the control group, and maintained firmness for a longer period, confirming the positive effect of coatings on the regulation of fruit transpiration and respiration.

During avocado ripening, an increase in oil content was observed, which was closely associated with the progressive loss of fruit moisture. This increase in lipid content is mainly related to a higher concentration of monounsaturated fatty acids, particularly oleic acid, which contributes to improved palatability and enhanced nutritional value of the fruit. Ref. [71] reported that the oleic acid content in avocado oil can vary significantly depending on cultivar and maturity stage, reaching concentrations of up to 71%. Concomitantly, the increase in oil content is accompanied by a decrease in fruit water content, resulting in a denser texture and higher nutritional value.

Pérez-Vergara et al. [72] demonstrated that oil content in avocados is directly influenced by the ripening process, highlighting the importance of this component in determining final fruit quality. In addition, the fatty acid profile undergoes changes during ripening, characterized by an increase in monounsaturated fatty acids, such as oleic acid, and a decrease in polyunsaturated fatty acids. These changes are driven by a complex metabolic process in which lipid catabolism is activated as glucose reserves become depleted. This process involves the β-oxidation of lipids, leading to the breakdown of triglycerides into glycerol and fatty acids and the generation of acetyl-CoA, which enters the Krebs cycle for energy production [72]. In this context, [64] emphasize that modifications in lipid composition during ripening are critical for increasing oil content and improving the sensory characteristics of avocados.

Gonçalves et al. [71] reported that, under ambient temperature storage conditions, weight loss in avocados increases progressively over time. As the fruit approaches ripening, the respiration rate increases, accelerating water loss and activating lipid metabolism, potentially affecting fruit quality. Similarly, [64] highlighted that weight loss is significantly greater at higher temperatures compared with refrigerated storage. This behavior is consistent with the findings of [73], who associated the reduction in pulp moisture with an increase in oil and fatty acid content, thereby enhancing fruit palatability.

Exposure to uncontrolled temperatures promotes dehydration, leading to tissue damage and a reduction in firmness, as explained by Pérez-Vergara et al. [72]. These authors also emphasized that the application of edible coatings can effectively reduce moisture loss, although it does not completely prevent metabolic degradation during storage. In the present study, weight losses of 30.89% and 12.97% were recorded after 30 days of storage for uncoated avocados stored at ambient temperature and coated avocados stored under refrigeration, respectively (Table 6). These results clearly demonstrate the protective effect of the edible coating when combined with low-temperature storage.

### 3.7. Sensory Attributes of Fuerte Avocado

Figure 5 presents the results of the sensory evaluation of Fuerte avocados coated with a film-forming solution based on purple corn flour biopolymers and ethanolic propolis extract (MMH 80%/EEP 20%), applied by the immersion method, as well as uncoated avocados stored at ambient temperature. Sensory attributes including overall acceptability, appearance, aroma, astringency, texture, and flavor were evaluated on days 0, 5, 10, 15, 20, 25, 30, and 35 of storage.

The results revealed significant differences in overall acceptability over storage time (*p* < 0.05). For coated avocados, panelists did not perceive significant sensory differences up to 30 days, whereas uncoated samples exhibited noticeable sensory deterioration from day 15 onward. The edible coating improved surface uniformity, reduced cracking, and prevented the development of visual defects, all of which are key factors influencing consumer acceptance. Nevertheless, as storage progressed, both coated and uncoated fruits showed a gradual decline in organoleptic quality, attributable to natural postharvest senescence processes.

Regarding appearance, no significant differences were observed up to 30 days in the coated avocados and up to 15 days in the control samples. The coating limited dehydration and loss of firmness, resulting in less wrinkling of the skin; however, the visual quality gradually declined with storage time. Regarding aroma, significant differences were recorded that intensified over time, becoming perceptible up to 30 days in the coated avocados and up to 15 days in the uncoated ones. Additionally, the panelists noted that the ethanolic propolis extract imparted a more pleasant aroma to the fruit. Subsequently, the aromatic quality decreased, primarily as a consequence of senescence. For astringency, significant differences were observed between treatments, with a progressive decrease throughout storage, possibly associated with the accumulation of fatty acids and the increase in dry matter content during ripening. Regarding texture, coated avocados exhibited greater palatability up to 30 days of storage, while uncoated samples maintained an acceptable texture only up to 15 days. In both cases, texture was negatively affected in later stages of storage due to senescence. Finally, significant differences were detected between treatments in the flavor attribute. Coated avocados maintained an acceptable flavor for up to 30 days, while control samples only maintained it for up to 15 days. In both cases, the sensory quality of the flavor progressively decreased over time, as a result of the metabolic processes associated with senescence during post-harvest storage.

It should be noted that chilling injury was not quantitatively evaluated in this study. Although refrigeration at 6 °C effectively extended the storage period of coated avocados and chilling injury was assessed indirectly through physicochemical, textural, and sensory changes, the quantification of specific physiological and cellular markers, such as electrolyte leakage or membrane integrity, would allow a more precise evaluation of cold-induced damage. In this regard, electrolyte leakage analysis is proposed as a relevant parameter for future studies aimed at further elucidating the effects of refrigerated storage on the quality of Fuerte avocado.

## 4. Conclusions

The physicochemical properties of propolis and purple corn flour complied with current international and Peruvian standards, confirming their quality and suitability as raw materials for the development of edible films. The physical and mechanical characterization of the formulated films, particularly those based on the combination of purple corn flour and ethanolic propolis extract, showed favorable results in terms of viscosity, pH, thickness, and tensile strength. However, films composed exclusively of propolis could not be obtained due to its predominantly lipidic nature, which limits its film-forming capacity.

The application of the combined edible coating (MMH 80%/EEP 20%) on Fuerte avocados by the immersion method proved effective in reducing weight loss, maintaining firmness, and preserving sensory quality during storage under both ambient and refrigerated conditions. Physicochemical and sensory evaluations confirmed that the coating improved appearance, gloss, and overall acceptability, extending the shelf life of avocados up to 35 days at ambient temperature and up to 72 days under refrigeration. The results indicate that the combination of purple corn flour and ethanolic propolis extract constitutes a promising natural biopolymeric system for the formulation of edible films and coatings. This approach offers high potential as a sustainable alternative to conventional food packaging, contributing to postharvest preservation and quality improvement of fresh horticultural products.

## Figures and Tables

**Table 1:** Summary of performance, advantages, and limitations of representative biopolymers used in edible film formulations.

Biopolymer/Technology	Typical Performance	Advantages	Limitations	References
Chitosan (CS)	Good oxygen barrier, moderate antimicrobial activity, good film-forming ability	Biodegradable, antimicrobial, good film-forming capacity	High water vapor permeability; mechanical fragility	[22,23]
Proteins (gelatin, casein, whey)	Good transparency, adhesion, and mechanical resistance	Excellent film-forming ability, biodegradable, good gas barrier	Sensitive to moisture; requires plasticizers	[24,25]
Polysaccharides (starch, pectin, CMC)	Good oxygen and oil barrier; chemical stability	Low cost, biodegradable, non-toxic	High water solubility; weak mechanical strength	[26]
Chitosan + plant extracts (polyphenols, propolis)	Strong antioxidant/antimicrobial activity; improved fruit shelf life	Enhanced functionality; high bioactive potential	Color changes; potential reduction in mechanical strength	[25,27]
Nanocellulose (CNF/SCNF)	High mechanical strength, good structural stability	Advanced biopolymer, biodegradable, excellent barrier properties	Requires chemical modification; higher cost	[28,29]

Note: The term “biopolymer technology” refers to the type of application of the biopolymer system, such as edible films or coatings used for postharvest preservation.

**Table 2:** Composition of MMH and MMH/EEP Film-Forming Solutions (%).

Materials	MMH 100%	MMH 80%/EEP 20%	MMH 90%/EEP 10%
Purple corn flour (MMH)	3.0	2.4	2.7
Ethanolic propolis extract (EEP)	-	0.6	0.3
Glycerol	2.0	2.0	2.0
70° Ethanol–water	95	95	95

Note: Film-forming solutions were prepared to a final volume of 100 mL. The concentrations of MMH, EEP, and glycerol are reported as % (*w*/*v*, g per 100 mL of solution). The volume of the 70% ethanol–water solvent was adjusted to account for the added solids.

**Table 3:** Physicochemical characterization of EEP and MMH for the development of biopolymer films.

Materials	Ash Content (%)	Moisture Content (%)	Insoluble Material (%)	Total Anthocyanin Content (TAC, mg Cyanidin-3-Glucoside/g Dry Sample)	pH
EEP	0.20 ± 0.02 ^a^	7.28 ± 0.72 ^b^	21.19 ± 2.15 ^a^	-	4.02 ± 0.02 ^b^
MMH	0.16 ± 0.02 ^b^	12.86 ± 0.61 ^a^	-	7.99 ± 1.72 ^a^	6.53 ± 0.25 ^a^

Note: TAC values are expressed as mg of cyanidin 3 glucoside equivalents per g of dry sample (mg C3G/g). TAC was determined only for MMH, as the ethanolic propolis extract (EEP) does not contain anthocyanins. Mean values ± standard deviation are reported. Different lowercase letters indicate significant differences (*p* < 0.05), according to Tukey’s post hoc test.

**Table 4:** Rheological parameters obtained from the Ostwald–de Waele model fitting and correlation analysis of the MMH and MMH/EEP film-forming solutions.

Formulation	rpm	γ (s^−1^)	τ (Pa)	Apparent Viscosity (Pa·s)	R^2^ (Ostwald–de Waele Model)
MMH 100% (Control)	10	12.23	11.90	0.07727	0.9728
	20	24.46	16.05	0.06560	
	30	36.69	16.59	0.04523	
	40	48.92	17.14	0.03504	
	50	61.15	17.85	0.02918	
MMH 80%/EEP 20%	10	12.23	24.11	0.1971	0.9513
	20	24.46	31.70	0.1296	
	30	36.69	35.24	0.1158	
	40	48.92	38.14	0.08432	
	50	61.15	41.25	0.05709	
MMH 90%/EEP 10%	10	12.23	16.36	0.1338	0.8796
	20	24.46	24.15	0.1187	
	30	36.69	26.25	0.06400	
	40	48.92	28.19	0.05216	
	50	61.15	30.14	0.04544	

Note: R^2^ corresponds to the coefficient of determination obtained from fitting the experimental data to the Ostwald–de Waele model. The model parameters (K and n) were not calculated, as the model was applied solely as a descriptive tool for comparative evaluation of flow behavior among formulations.

**Table 5:** Physical characteristics of MMH and EEP edible films.

Edible Films	pH	Solubility (%)	Thickness (mm)	Permeability (g.mm/h.m^2^Pa)
MMH 80%/EEP 20%	6.42 ± 0.03 ^b^	49.69 ± 0.58 ^c^	0.146 ± 0.010 ^c^	0.7238 ± 0.055 ^c^
MMH 90%/EEP 10%	6.48 ± 0.01 ^a^	52.52 ± 0.62 ^b^	0.204 ± 0.008 ^b^	1.0477 ± 0.065 ^b^
MMH 100% (control)	6.53 ± 0.01 ^a^	55.11 ± 0.56 ^a^	0.226 ± 0.014 ^a^	1.2492 ± 0.032 ^a^

Note: MMH: Purple corn flour; EEP: Ethanolic propolis extract. Different letters within a column indicate significant differences (*p* < 0.05).

**Table 6:** Physicochemical characteristics of Fuerte avocado with and without edible coating, stored for up to 35 days at 20 °C and 72 days at 6 °C.

Formulations	Titratable Acidity (% Citric Acid)	Moisture Content (%)	Oil Content (%)	Texture (Penetration, 0.1 mm)	pH	Weight Loss (%)
Day 0						
MMH/EEP (20 °C)	0.04 ± 0.00 q	84.22 ± 0.97 t	9.62 ± 0.24 a	1.71 ± 0.05 a	6.13 ± 0.01 a	-
Control (20 °C)	0.04 ± 0.00 q	84.74 ± 0.29 t	9.32 ± 0.59 a	1.71 ± 0.06 a	6.13 ± 0.01 a	-
MMH/EEP (6 °C)	0.04 ± 0.00 q	84.56 ± 0.41 t	9.43 ± 0.55 a	1.71 ± 0.01 a	6.12 ± 0.01 a	-
Control (6 °C)	0.04 ± 0.00 q	84.74 ± 0.29 t	9.32 ± 0.59 a	1.71 ± 0.02 a	6.12 ± 0.01 a	-
Day 5						
MMH/EEP (20 °C)	0.03 ± 0.00 op	77.68 ± 0.29 r	12.95 ± 1.02 cde	2.42 ± 0.02 defg	6.24 ± 0.01 cd	3.48 ± 0.02 bc
Control (20 °C)	0.03 ± 0.00 lmn	72.97 ± 0.57 op	15.20 ± 0.23 efg	7.31 ± 0.04 l	6.32 ± 0.01 f	7.01 ± 0.04 f
MMH/EEP (6 °C)	0.03 ± 0.00 op	82.72 ± 0.30 t	10.76 ± 0.88 abc	1.86 ± 0.02 ab	6.13 ± 0.01 a	1.70 ± 0.02 a
Control (6 °C)	0.03 ± 0.00 pq	83.00 ± 0.13 t	10.56 ± 0.75 ab	1.91 ± 0.01 ab	6.15 ± 0.01 ab	1.94 ± 0.02 a
Day 10						
MMH/EEP (20 °C)	0.03 ± 0.00 klm	72.38 ± 0.47 o	17.53 ± 0.83 gh	3.17 ± 0.22 i	6.36 ± 0.01 g	4.68 ± 0.03 d
Control (20 °C)	0.02 ± 0.00 d	58.00 ± 0.41 f	23.18 ± 0.37 jklm	11.43 ± 0.17 o	6.50 ± 0.02 i	11.98 ± 0.04 j
MMH/EEP (6 °C)	0.03 ± 0.00 op	80.26 ± 0.12 s	12.52 ± 0.41 bcd	1.96 ± 0.04 abc	6.15 ± 0.01 ab	3.17 ± 0.04 b
Control (6 °C)	0.03 ± 0.00 op	80.12 ± 0.27 s	12.13 ± 0.83 bcd	2.25 ± 0.03 abc	6.17 ± 0.01 b	3.73 ± 0.03 c
Day 15						
MMH/EEP (20 °C)	0.03 ± 0.00 hij	65.87 ± 0.84 kl	20.52 ± 1.24 i	6.47 ± 0.20 k	6.42 ± 0.01 h	10.59 ± 0.04 i
Control (20 °C)	0.02 ± 0.00 c	54.66 ± 0.54 e	30.82 ± 0.49 pqr	24.34 ± 0.22 qr	6.52 ± 0.01 i	15.98 ± 0.04 n
MMH/EEP (6 °C)	0.03 ± 0.00 no	77.28 ± 0.70 r	14.50 ± 1.23 fgh	2.18 ± 0.02 bcd	6.22 ± 0.01 c	6.30 ± 0.03 e
Control (6 °C)	0.03 ± 0.00 mn	76.66 ± 1.13 r	16.26 ± 0.86 fgh	2.54 ± 0.02 defg	6.22 ± 0.01 c	7.49 ± 0.02 g
Day 20						
MMH/EEP (20 °C)	0.02 ± 0.00 f	62.48 ± 0.26 ij	22.97 ± 0.43 jklm	8.17 ± 0.10 m	6.49 ± 0.01 i	14.21 ± 0.05 l
Control (20 °C)	0.01 ± 0.00 b	50.44 ± 0.30 d	35.57 ± 0.16 s	40.82 ± 0.18 x	6.59 ± 0.02 j	19.51 ± 0.06 r
MMH/EEP (6 °C)	0.03 ± 0.00 mn	75.89 ± 1.09 qr	16.03 ± 0.55 fgh	2.27 ± 0.01 cde	6.23 ± 0.01 cd	7.84 ± 0.02 g
Control (6 °C)	0.03 ± 0.00 klm	72.91 ± 0.23 op	18.02 ± 0.34 h	2.64 ± 0.02 efgh	6.27 ± 0.01 de	9.55 ± 0.02 h
Day 25						
MMH/EEP (20 °C)	0.02 ± 0.00 d	58.41 ± 0.47 fg	23.24 ± 1.11 klm	10.45 ± 0.27 n	6.51 ± 0.01 i	17.47 ± 0.12 p
Control (20 °C)	0.01 ± 0.00 a	48.82 ± 1.01 cd	38.27 ± 1.16 t	40.77 ± 0.02 x	6.60 ± 0.01 j	25.28 ± 0.03 u
MMH/EEP (6 °C)	0.03 ± 0.00 klm	74.64 ± 0.34 pq	17.43 ± 0.99 gh	2.36 ± 0.02 def	6.24 ± 0.01 cd	9.42 ± 0.03 h
Control (6 °C)	0.03 ± 0.00 ijk	71.02 ± 0.33 no	20.87 ± 0.39 ijk	2.86 ± 0.02 hi	6.32 ± 0.01 f	11.72 ± 0.25 j
Day 30						
MMH/EEP (20 °C)	0.02 ± 0.00 c	53.50 ± 0.41 e	29.81 ± 0.30 op	25.89 ± 0.15 s	6.59 ± 0.02 j	19.58 ± 0.03 r
Control (20 °C)	0.01 ± 0.00 a	44.42 ± 1.24 b	41.30 ± 0.52 u	40.83 ± 0.02 xy	6.68 ± 0.01 l	30.89 ± 0.61 x
MMH/EEP (6 °C)	0.03 ± 0.00 jkl	70.99 ± 0.22 no	20.81 ± 0.16 ij	2.63 ± 0.02 bcde	6.28 ± 0.01 de	12.97 ± 0.02 k
Control (6 °C)	0.03 ± 0.00 hi	67.52 ± 0.50 lm	23.39 ± 0.52 lm	5.89 ± 0.03 j	6.41 ± 0.01 h	16.01 ± 0.02 n
Day 35						
MMH/EEP (20 °C)	0.01 ± 0.00 a	50.45 ± 0.23 d	33.17 ± 0.76 r	40.49 ± 0.48 x	6.65 ± 0.03 kl	20.26 ± 0.13 s
Control (20 °C)	0.01 ± 0.00 a	38.19 ± 0.49 b	44.24 ± 0.87 w	40.90 ± 0.02 y	6.74 ± 0.02 m	34.61 ± 0.07 z
MMH/EEP (6 °C)	0.03 ± 0.00 ij	69.40 ± 0.43 mn	21.77 ± 1.03 ijkl	2.67 ± 0.01 fgh	6.30 ± 0.01 ef	14.81 ± 0.02 m
Control (6 °C)	0.03 ± 0.00 gh	63.68 ± 1.47 j	25.35 ± 0.47 mn	7.52 ± 0.02 l	6.52 ± 0.01 i	18.60 ± 0.02 q
Day 40						
MMH/EEP (20 °C)	-	-	-	-	-	-
Control (20 °C)	-	-	-	-	-	-
MMH/EEP (6 °C)	0.03 ± 0.00 hi	67.38 ± 0.29 l	22.64 ± 0.83 ijkl	2.77 ± 0.02 gh	6.33 ± 0.01 fg	16.90 ± 0.08 o
Control (6 °C)	0.02 ± 0.00 fg	60.99 ± 0.58 hi	27.44 ± 0.42 no	19.88 ± 0.03 p	6.61 ± 0.01 jk	21.20 ± 0.02 t
Day 48						
MMH/EEP (20 °C)	-	-	-	-	-	-
Control (20 °C)	-	-	-	-	-	-
MMH/EEP (6 °C)	0.03 ± 0.00 gh	64.42 ± 0.33 jk	25.11 ± 0.82 mn	6.02 ± 0.03 j	6.61 ± 0.00 j	21.48 ± 0.03 t
Control (6 °C)	0.02 ± 0.00 d	54.93 ± 0.25 e	30.71 ± 0.17 op	24.02 ± 0.03 q	6.66 ± 0.01 l	27.07 ± 0.05 w
Day 56						
MMH/EEP (20 °C)	-	-	-	-	-	-
Control (20 °C)	-	-	-	-	-	-
MMH/EEP (6 °C)	0.03 ± 0.00 fg	60.29 ± 0.38 gh	26.69 ± 0.30 n	7.99 ± 0.03 m	6.66 ± 0.01 l	27.27 ± 0.04 w
Control (6 °C)	0.02 ± 0.00 d	50.13 ± 0.16 d	35.67 ± 0.46 s	27.92 ± 0.03 t	6.73 ± 0.01 m	34.13 ± 0.05 y
Day 64						
MMH/EEP (20 °C)	-	-	-	-	-	-
Control (20 °C)	-	-	-	-	-	-
MMH/EEP (6 °C)	0.02 ± 0.00 e	57.57 ± 0.29 f	29.27 ± 0.28 op	24.52 ± 0.02 r	6.67 ± 0.01 l	34.13 ± 0.05 y
Control (6 °C)	0.02 ± 0.00 c	47.39 ± 0.18 c	38.76 ± 0.81 t	30.14 ± 0.02 u	6.79 ± 0.01 n	38.19 ± 0.07 ’b
Day 72						
MMH/EEP (20 °C)	-	-	-	-	-	-
Control (20 °C)	-	-	-	-	-	-
MMH/EEP (6 °C)	0.02 ± 0.00 d	53.61 ± 0.42 e	32.63 ± 0.38 qr	27.93 ± 0.02 t	6.67 ± 0.03 l	36.50 ± 0.07 ’a
Control (6 °C)	0.02 ± 0.00 bc	44.43 ± 1.31 b	39.09 ± 1.46 tu	32.21 ± 0.01 w	6.86 ± 0.01 q	42.32 ± 0.03 ’c

Note: Different lowercase letters within the same column indicate significant differences among treatments at the same storage time (*p* < 0.05), according to Tukey’s post hoc test. Comparisons were performed independently for each storage day; therefore, letters should not be compared across different days. Day 0 values correspond to the initial condition and were not subjected to statistical comparison among treatments.

## Data Availability

The original contributions presented in this study are included in the article. Further inquiries can be directed to the corresponding author.
